# A biocompatible reverse thermoresponsive polymer for ocular drug delivery

**DOI:** 10.1080/10717544.2019.1587042

**Published:** 2019-03-24

**Authors:** Asitha Balachandra, Elsa C. Chan, Joseph P. Paul, Sze Ng, Vicki Chrysostomou, Steven Ngo, Roshan Mayadunne, Peter van Wijngaarden

**Affiliations:** a CSIRO Molecular Science & Health Technologies, Victoria, Australia;; b Centre for Eye Research Australia, Royal Victorian Eye and Ear Hospital, Melbourne, Australia;; c Ophthalmology, Department of Surgery, University of Melbourne, Melbourne, Australia

**Keywords:** Ocular drug delivery, anti-vascular endothelial growth factor, intravitreal injection, reverse thermoresponsive polymer, macular degeneration

## Abstract

Age-related macular degeneration (AMD) is a leading cause of vision loss, the treatment of which may require monthly intravitreal injections. This is a burden on patients and health services, and new delivery modalities that reduce injection frequency are required. To that end, we investigated the suitability of a novel reverse thermoresponsive polymer (RTP) as an ocular drug-delivery vehicle. In this work, we detail the structure and synthesis of a novel RTP, and determine drug release curves for two drugs commonly used in the treatment of AMD, bevacizumab and aflibercept. Biocompatibility of the RTP was assessed *in vitro* in human and rat cell lines and *in vivo* following intravitreal injection in rats. Bevacizumab demonstrated a more appropriate release profile than aflibercept, with 67% released within 14 days and 78% released in total over a 183-day period. No toxic effects of RTP were seen in human or rat cells in up to 14 days of co-culture with RTP. Following intravitreal injection, intraocular pressure was unaffected by the presence of RTP and no changes in retinal function or structure were observed at 1 week or 1 month post-injection. RTP injection did not cause inflammation, gliosis or apoptosis in the retina. This work demonstrates the potential suitability of the novel RTP as a sustained-release vehicle for ocular drug delivery for anti-neovascular therapies. Optimization of polymer chemistry for optimal drug loading and release is needed.

## Introduction

Age-related macular degeneration (AMD) is the leading cause of blindness in Australians over the age of 60 years. In a subset of patients with the most aggressive form of the disease abnormal blood vessels grow beneath the retina and the associated leakage of fluid and blood reduces vision and predisposes to scarring and permanent retinal damage (Shao et al., 2016). The treatment of this form of the disease has been revolutionized by the development of drugs targeting vascular endothelial growth factor (VEGF) (Schachat, 2013), which is produced by the diseased retina and which drives the development of abnormal blood vessels. Anti-VEGF drugs are delivered by injection into the eye as often as monthly for indefinite periods of time (Solomon et al., 2014). Clinical services are already straining under the demand for this treatment, which is predicted to increase (Prenner et al., 2015; Saxena et al., 2016). This therapy has also recently become the mainstay of treatment for diabetic macular edema – a leading cause of vision loss in working-age adults (Keel et al., 2017). The need for frequent eye injections imposes significant social costs upon patients and exposes them to the cumulative risk of sight-threatening complications of the procedure (Ramsey et al., 2014; Nuzzi and Tridico, 2015; Eadie et al., 2017). In light of this increasing demand, it is necessary to develop a sustained-release intraocular drug delivery vehicle to reduce the frequency of eye injections.

In recent years, several polymers have been investigated for use as a vehicle for sustained drug delivery in the eye, including microspheres (Andres-Guerrero et al., 2015; Aramwit et al., 2015), micelles (Mayol et al., 2014) and hydrogels (Ahmed, 2015). Among these compounds, hydrogels have received much attention due to their versatile properties (Ahmed, 2015; Kirchhof et al., 2015). Hydrogels can absorb a large volume of water (up to 1000 times their dry weight) and are able to alter their physical properties, for example transitioning from gel-to-solution, or solution-to-gel, in response to external stimuli including pH, temperature, ionic strength and magnetic field (Hernandez et al., 2010).

These properties make hydrogels intriguing candidates for use as sustained release vehicles. In the case of temperature-responsive polymers, a drug added to the hydrogel in its liquid state becomes incorporated into the gel as temperature is increased (Park et al., 2013; Rauck et al., 2014). Over time, drug is released from the polymer gel, providing a sustained delivery mechanism. In this way, therapeutic compounds may be added to a hydrogel and injected into the eye in the liquid state, rapidly forming a gel on exposure to body temperature, encapsulating the drug and enabling its sustained intraocular release.

An important consideration for the medical use of hydrogels is biodegradability. The two most widely investigated thermoresponsive polymers are based on an acrylamide polymer backbone, such as poly(n-isopropyl acrylamide) [p(NIPAM)], Poloxamers (ICI, Cleveland, UK) and poly ether backbone, such as Pluronic F127 by BASF (Ludwigshafen, Germany). These polymers are highly biostable, often necessitating the surgical removal of the expended residues upon completion of drug release (Morley et al., 1995; Dumortier et al., 2006; Alhalafi, 2017). In order to minimize surgical interventions, use of a biodegradable polymer, which would break down over time and not require removal, is preferable. As such, the toxicity not only of the polymer, but also of its constituent monomers, must be addressed. This is a potential concern and reason to avoid the use of an acrylamide backbone, as acrylamide monomers are known to be toxic for *in-vivo* applications (Imai and Kitahashi, 2014; Duan et al., 2015).

Of thermoresponsive polymers that are reported to be biodegradable, particularly those with polylactic-*co*-polyglycolic acid backbones, most are structured to have linear polymeric backbones, resulting in a loosely bound gel structure due to weak intermolecular attractions and entanglements with less solid content. This prevents the long term encapsulation of drugs within the gel, characterized by initially high burst release and faster ongoing release rates, thereby reducing the window of release period.

In this study, we describe a novel reverse thermoresponsive polymer (RTP) that takes up and slowly releases anti-VEGF agents, and possesses *in vitro* and *in vivo* biocompatibility, with potential utility as a novel sustained release intraocular drug delivery vehicle.

## Methods

### Animal ethics and husbandry

All *in vivo* procedures were performed in accordance with the ARVO Statement for the Use of Animals in Ophthalmic and Vision Research. All procedures were approved by the institutional animal care and use committee (AMREP animal ethics committee protocol E1615/2015). Adult male Long Evans rats sourced from ARC (Murdoch, WA, Australia) were used in this study. Animals were housed in temperature-controlled cages (23 °C) in a 12-hour light-dark cycle with *ad libitum* access to food and water.

### Synthesis of reverse thermoresponsive polymer: PE-LA-CL (75:25)-PEG (350) 3500 MW – TRP with four arms

#### STEP A

Pentaerythritol (PE) (3.2131 g, 1 mole), DL-Lactic acid (42.5177 g, 18 moles) and ε-Caprolactone (16.1622 g, 6 moles) were heated in a round bottom flask 160–170 °C in the presence of tetrahydrofuran (THF), 250 mL and 1.0 g of p-toluenesulphonic acid monohydrate. The reaction mixture was allowed to stir for 3 days at reflux and ambient pressure. The water generated was collected using a Dean-Stark apparatus. The solvent was decanted and the reaction mixture concentrated using a rotary evaporator and the residual solvent removed under high vacuum to produce a slightly yellow transparent product (∼80% yield) (Theoretical MW – 2118.43, GPC MW as observed: Mn 2766, Mw 3469, Mp 3356, Mz 4266, PD 1.25).

#### STEP B: Functionalization of polyester

The polyester polyol PE-LA-CL (75:25) (0.5 × 10^−3^ moles) was dissolved in dry dichloromethane (DCM) (15 mL) in a round bottom glass with a magnetic stirrer bar and hexane diisocyanate (HDI) (20 × 10^−3^ moles, 10 fold excess) added at room temperature. The reaction mixture was stirred for 4 h and 10 mg of dibutyltin dilaurate (DBTL) added. The mixture was stirred at ambient temperature overnight. The product was precipitated into dry n-heptane (1500 mL), decanted, and the polymer residue immediately re-dissolved in DCM for the next functionalization step.

#### STEP C: Addition of PEG-OCH3 350 MW

The HDI functionalized polyester polyol was dissolved in dry DCM (15 mL) and pre-dried monomethyl PEG-O-H (3 × 10^−3^ moles, 1.5 equivalents) added at room temperature. The reaction mixture was stirred for 4 h followed by the addition of the catalyst dibutyltin dilaurate (DBTL) (10 mg). The solution was allowed to stir over night at room temperature. The polymer product was precipitated into n-heptane (1000 mL), the solvent decanted, the precipitated polymer re-dissolved in a minimum of DCM, transferred to a round bottom flask, and the solvent removed using a rotary evaporator. The residual solvents in the product polymers were removed by high vacuum to obtain the crude final polymer.

#### STEP D: purification procedure

The crude product polymers were dissolved in de-ionized water below 10 °C. Upon complete dissolution the mixture was heated to 60 °C to precipitate and isolate polymers from solution. This precipitation was conducted three times to isolate the purified polymer product.

#### STEP E: procedure for the preparation of aqueous polymer solutions

Polymer solutions for release of active agents were prepared by dissolving purified polymer in distilled water at 5–10 °C overnight with constant mixing. The active agent was separately dissolved in distilled water and added to the completely dissolved polymer solution, mixed in with a fine spatula and a vortex mixer to afford a uniform solution.

### In vitro release of anti-VEGF agents from the thermoresponsive polymer

The stock polymer solutions for release of bevacizumab (Genentech) and aflibercept (Regeneron Pharmaceuticals) a monoclonal antibody and antibody-receptor fusion protein, respectively, were prepared using the intravitreal injection solutions available from the suppliers (25 mg/mL and 40 mg/mL, respectively) and by dissolving each with the purified polymer in liquid phase (as above, at ≤10 °C) with or without phosphate buffer solution to afford a 1.8% solution of the active agent. Details of reagent masses can be found in Supplementary materials Tables S1 (bevacizumab) and S2 (aflibercept)

The polymer:anti-VEGF solutions (125 mg) were placed at the bottom of glass sample vials and incubated at 37 °C for 2 h. PBS (pH = 7.0) (37^0^ C, 0.5 mL) was added to each sample vial while maintaining temperature. The polymer:active agent compound remained at the bottom of the glass vial as a gel while the added PBS remained above the polymer gel. The samples were placed in an incubator 37 °C and shaken at 50 RPM to provide the best chance of release of the active agent from the polymer gel mixture and eliminate any localized concentration effects in the release solution over the period of interest. At each sampling time point (0, 1 3, 7, 14, 60 and 183 days) 0.25 mL of the release solution was withdrawn carefully while maintaining the sample at the same temperature and the same volume of fresh PBS solution (37 °C) was added. Every effort was taken to prevent changes in temperature.

The total soluble protein in the withdrawn release solution was quantified with a QuantiPro Bicinchoninic acid (BCA) protein assay kit (Sigma-Aldrich) in triplicate at each time point. The replicates at each time point were averaged and the released protein was quantified using a plate reader spectrophotometer (Bio-Tek PowerWave XS) against the mean absorbance vs concentration calibration curves evaluated separately on each day. For both aflibercept and bevacizumab calibration curves were generated on each day for the following concentrations (1 mg/mL, 0.4 mg/mL, 0.2 mg/mL, 0.1 mg/mL, 0.03 mg/mL, 0.02 mg/mL, 0.01 mg/mL, 0.005 mg/mL, 0.0005 mg/mL, 0 mg/mL).

### Cell culture and cell viability

Human neuroblastoma (SHSY-5Y) cells and rat fibroblasts (R-12) were obtained from ATCC (Manassas, VA, USA) and maintained in Dulbecco’s Modified Eagle Medium (DMEM, Invitrogen, USA) supplemented with 10% fetal calf serum, penicillin (100 U/mL, Invitrogen) and streptomycin (100 µg/mL, Invitrogen) at 37 °C in 5% CO_2_. RTP (15 μl) was added to each well of a 6-well plate and solidified in an incubator at 37 °C prior to seeding of the wells with cells (20,000 cells) in DMEM. The number of viable cells was determined using Trypan blue staining and evaluated by Countess^®^ Automated Cell Counter (C10227, Invitrogen, USA). Experiments were performed in triplicate in parallel with controls on days 7, 14, 21 and 28 for rat fibroblasts and on days 1, 3, 6 and 11 for SHSY-5Y cells.

### Intravitreal injection

Male Long-Evans rats (7–8 weeks) were anesthetized with an intraperitoneal injection of ketamine (80 mg/kg) and xylazine (10 mg/kg). Residual eye reflexes were blocked by topical application of oxybuprocaine hydrochloride 0.4% (Alcon Laboratories, Fort Worth, Texas, USA) to the corneal surface. The pupil was dilated with a single drop of both 0.5% phenylephrine (Alcon) and 0.5% tropicamide (Alcon). A sterile 34 G needle was inserted into the vitreous cavity behind the limbus and RTP (5 µL, RTP group) or saline (5 µL, control group) was slowly injected using a Hamilton syringe and microsyringe pump (at 200 nL/s over 25s). In order to control for potential confounds from physical trauma as a result of the injection procedure, contralateral eyes received a sham procedure in which a needle was inserted as above but no injection took place. Topical chloramphenicol was administered after the injection to prevent infection.

### Clinical examination

Clinical examination was performed before and immediately after, and at 1 day, 1 week and 4 weeks after injection. Examinations included assessment of general health, intraocular pressure (IOP) measurement and ocular slit lamp examination. The IOP was measured with a rebound tonometer (TonoVet; Icare, Helsinki, Finland). Slit lamp examination of ocular structures including conjunctiva, iris, lens, sclera, cornea, retina and optic nerve was performed after dilating the eyes with tropicamide (0.5%). Retinal photography was also performed in anesthetized rats using the Micron III rodent imaging suite (Phoenix Research Labs, Pleasanton, CA, USA) immediately after injection and at 1 week and 1 month after injection.

### Electroretinography

The full-field flash electroretinogram (ERG) was used to assess retinal function in dark-adapted anesthetized animals as previously described (Kong et al., 2011). Signals were recorded with a 4 mm platinum wire loop active electrode contacting the cornea, and a gold cup reference electrode placed in the mouth. A subdermal needle electrode inserted at the base of the tail acted as a ground electrode. Retinal responses to a series of stimulus intensities (from −5.92 to 2.22 log cd.s/m^2^) were recorded simultaneously from both eyes over a period of 30 min. ERG responses from each animal were recorded 1 week before intravitreal injection (as baseline) and 1 and 4 weeks after injection.

### Tissue processing and histology

Subsets of animals were euthanized at one week (*n* = 5) and 1 month (*n* = 5) after injection for histology and immunohistochemistry. Eyes were harvested, and fixed in 4% paraformaldehyde for 1 h, followed by an overnight incubation in sucrose (15%). OCT-embedded sections (12 µm) were then stained with hematoxylin and eosin to assess morphology.

### Immunohistochemical detection of macrophages, microglia and gliosis

OCT-embedded sections (12 µm) were blocked with 10% goat serum with 0.3% Triton X-100 for 1 h at room temperature. Sections were then incubated overnight (4 °C) with an antibody with specificity for ionized calcium binding adaptor molecule 1 (Iba-1) expressed by macrophages and microglia (rabbit anti-mouse polyclonal Iba-1 antibody, 0.5 g/mL, catalog no. 019-19741, Wako Laboratory Chemicals) and an antibody for glial cell activation (mouse anti-rat glial fibrillary acidic protein, GFAP, Sigma). Sections then were incubated for 1 hour with secondary antibodies (Alexa Fluor 488 goat anti-mouse antibody, 0.01 mg/mL, A21236, Alexa Fluor 594 goat anti-rabbit antibody, 0.01 mg/mL, A11037). Sections were mounted in Dako fluorescent mounting media. For evaluation of Iba-1 immunopositive cells and retinal thickness, measurements were made in one central (within 200 µm of the optic nerve) and two peripheral areas of retina (superior and inferior to the optic nerve) to generate an average measurement per eye. The number of Iba-1 + ve cells was normalized to the retinal area, and retinal layer thicknesses were normalized to total retinal thickness.

### Apoptotic cell counting

The extent of cell apoptosis in OCT-embedded sections (12 µm) was assessed with an in situ cell detection kit (Catalog no. 12156792910, Roche, Germany) according to manufacturer’s instructions. For determination of TUNEL-positive apoptotic cells in the retina, measurement was made in each retinal layer in two whole eye cryosections (12 µm thick, 120 µm apart) obtained from one central and two peripheral regions (as for retinal thickness measurement) and an average value per eye was calculated.

### Statistical analysis

Values are expressed as mean ± S.E.M. Mean values were compared using one-way analysis of variance (ANOVA) followed by *post-hoc* Tukey analysis. A value of *p* < .05 was regarded as statistically significant.

## Results

### 

#### Release characteristics of RTP

The putative structure of the RTP is depicted in Figure 2(A). Release of aflibercept and bevacizumab from the RTP was asssesed in vitro in conditions designed to mimic the intraocular environment. Figure 2(B) depicts drug release over the first 23 days and Figure 2(C) shows release over the duration of the 183 day experiment.

**Figure 1.**
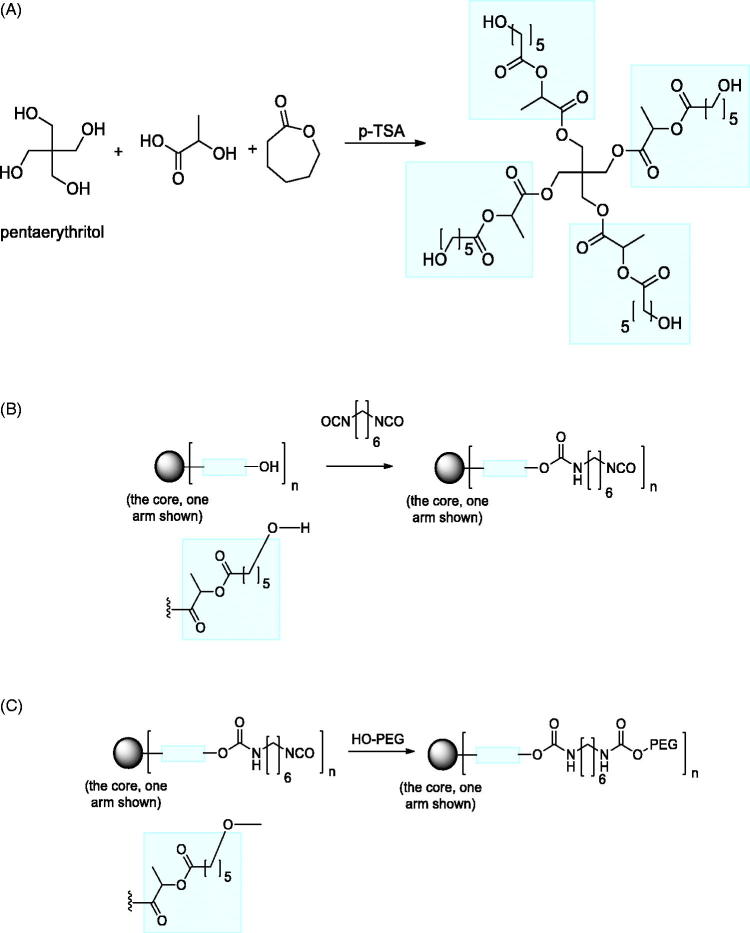
Steps in the preparation of a typical reverse thermoresponsive polymer. (A) Preparation of the multi-arm core polyester. (B) Functionalisation of the terminal ends of the arms of the core polyester. (C) Addition of the hydrophilic block ethylene glycol polymer to the terminal ends of the multi-arm polymer.

**Figure 2.**
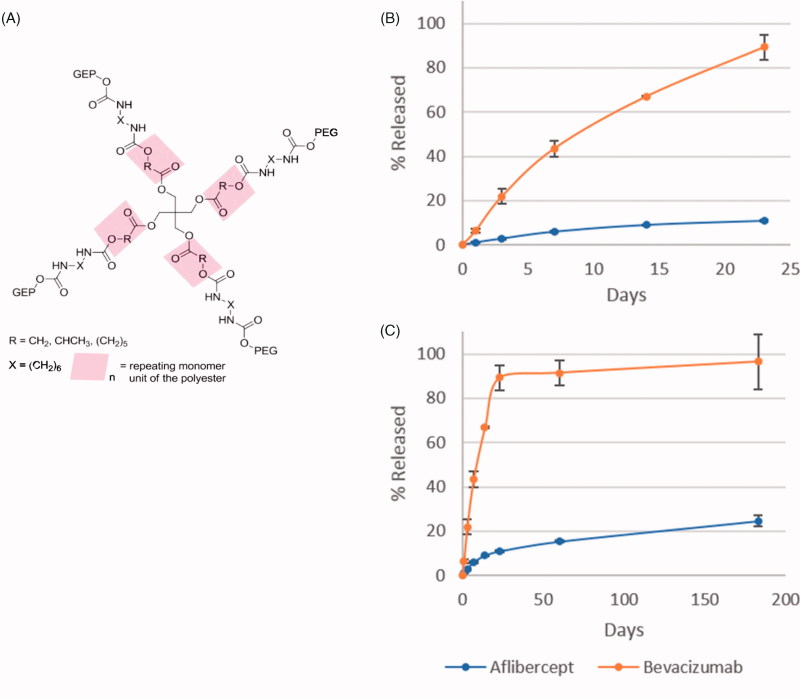
Structure and release profiles of RTP. Panel A depicts the 4-armed structure of the RTP, while B and C show the short- and long-term release profiles of aflibercept (blue) and bevacizumab (orange) respectively. Data shown as mean ± SD.

#### RTP does not affect the viability of rat and human cell lines

**Figure 3.**
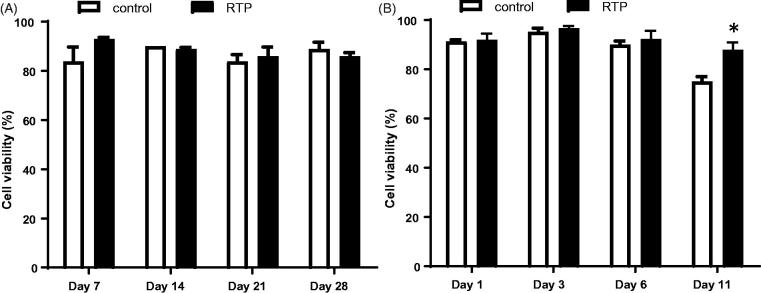
*In vitro* biocompatibility of the reverse thermoresponsive polymer (RTP) with (A) rat fibroblasts and (B) human SHSY-5Y cells. RTP had no adverse effect on cell viability in either cell line. Experiments were performed in triplicate except for day 14 and day 11 (in duplicate) in parallel with controls. *p < .05 in comparison to control on day 11.

To evaluate *in vitro* biocompatibility of RTP, rat R12 fibroblasts and human SHSY-5Y cells were incubated with RTP (15 µL) for 28 and 11 days respectively. RTP without anti-VEGF loading was used for all subsequent experiments. The presence of RTP in the culture medium did not adversely affect viability of rat fibroblasts for up to 28 days (*p* = .56, *n* = 2–3), or human neuronal (SHSY-5Y) cells for up to 11 days (*p* = .25, *n* = 2–3) (Figure 3). RTP treated SHSY-5Y cells show a slightly higher cell viability than control at day 11 (75 ± 4% vs 88 ± 4% in RTP; *p* < .05, *n* = 2–3).

#### RTP injection in rat eyes does not alter intraocular pressure (IOP) or retinal morphology

**Figure 4.**
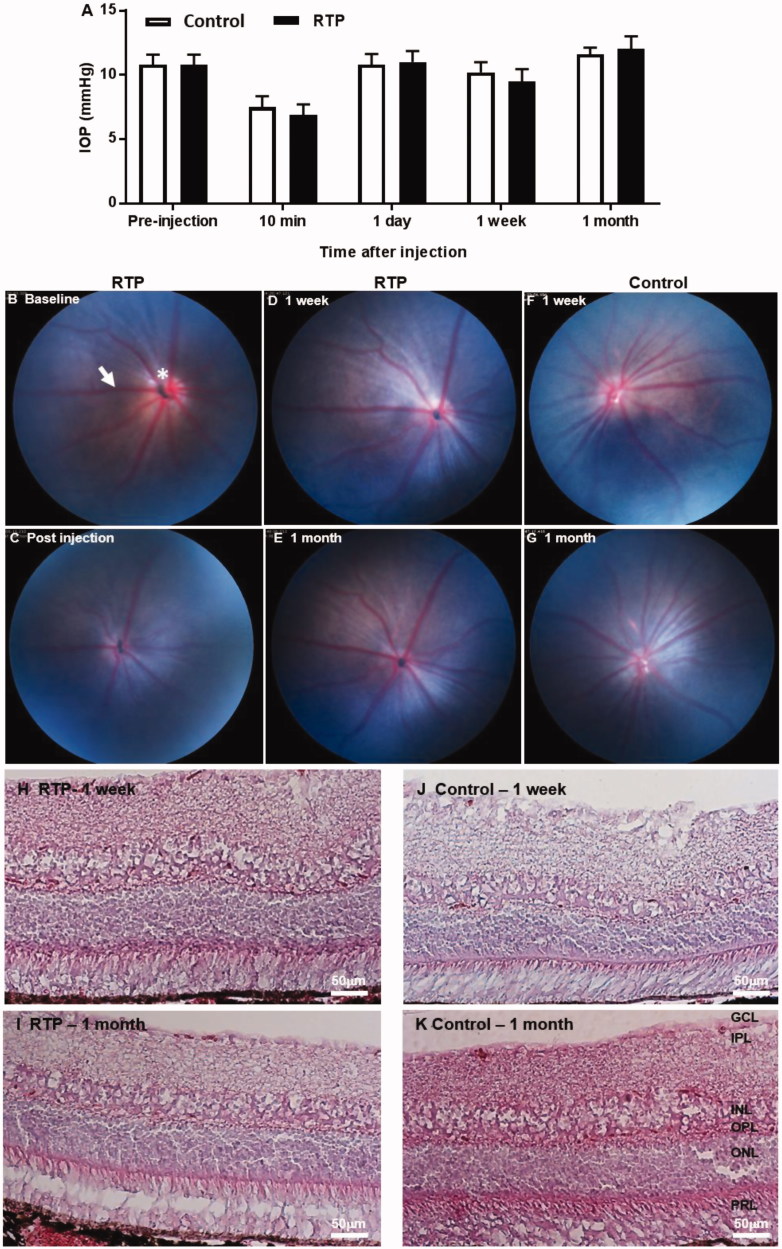
*In vivo* biocompatibility of the RTP following intravitreal injection in rats. (A) IOP was measured before and after control and RTP intravitreal injections. Data shown as mean ± SEM. Retinal photographs of control (B, C) or RTP (5 μl, D, E, F, G) injected rat eyes. A retinal blood vessel is indicated by an arrow and the optic nerve head is indicated by an asterisk in (D). Hematoxylin-eosin stained retinal sections from control (H, I) and RTP (J, K) injected rats. No intraocular abnormalities were evident in eyes receiving RTP or control injection. Retinal layers are labeled: GCL: ganglion cell layer; IPL: inner plexiform layer; INL: inner nuclear layer; OPL: outer plexiform layer; ONL: outer nuclear layer; PRL: photoreceptor layer.

Drug-free RTP or an equal volume of PBS (control) was injected intravitreally in rat eyes. Measurement of IOP was performed at baseline and then once or twice per week after injection. As expected, both control and RTP injection decreased IOP measurements at 10 min post injection (Figure 4), the result of efflux of a small amount of vitreous humor after the injection procedure. Pressure returned to pre-injection levels at 1 day after injection. The presence of RTP in the eye did not affect IOP at up to 1 month after administration (*p* = .80). Retinal photographs were acquired prior to and immediately after injection, as well as at 1 week and 1 month after injection (Figure 4). No evidence of retinal abnormalities was seen in control or RTP injected rats (Figure 4(D–G)). Histological examination of retinal sections from rats receiving control or RTP injections were comparable with no evidence of cell loss or injury (Figure 4(H–K)).

#### RTP injection does not cause intraocular inflammation

**Figure 5.**
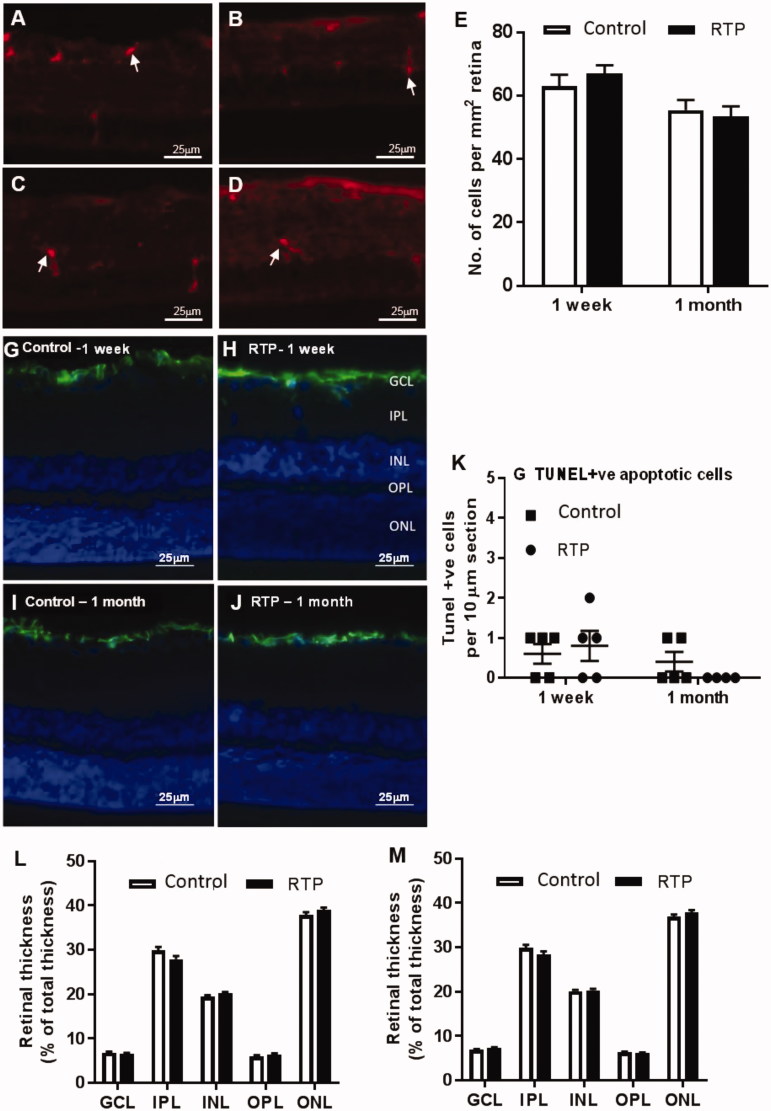
Detection of Iba-1, GFAP, and TUNEL positive cells in retinal sections. Representative retinal images at 1 week (A, B) and 1 month (C, D) after injection. (E) No significant differences in the number of Iba-1 immunopositive cells in RTP and control injected eyes at 1 week (*p* = .40, *n* = 5) and 1 month (*p* = .87, *n* = 5). Representative GFAP immunohistochemistry images at 1 week (G,H) and 1 month (I,J) after injection. There was no difference in the number of apoptotic cells (K) between control and RTP treatment at 1 week (*p* = .67, *n* = 5) and 1 month (*p* = .19, *n* = 4–5). Retinal layer thickness normalized to the total retinal thickness at 1 week (L) and 1 month (M). GCL: ganglion cell layer; IPL: inner plexiform layer; INL: inner nuclear layer; OPL: outer plexiform layer; ONL: outer nuclear layer. Data shown as mean ± SEM.

To investigate the effect of RTP injection on inflammatory activity in the retina, we examined the expression of Iba-1 immunopositive microglia and macrophages in retinal sections (Figure 5) at 1 week and 1 month post injection. Iba-1 immunopositive cells were sparsely distributed in the retina in both control (Figure 5(A,C)) and RTP injected rats (Figure 5(B,D)), and predominantly displayed a ramified, inactive morphology (Nimmerjahn et al., 2005). RTP injection did not influence the accumulation of Iba-1 immunopositive cells in the retinas at 1 week (*p* = .40, *n* = 5) and 1 month post injection (*p* = .87, *n* = 5; Figure 5(E)).

#### RTP injection does not induce retinal gliosis or neurodegeneration

To investigate the effect of RTP injections on inflammation and glial cell activation in the retina, the expression of GFAP was evaluated in retinal sections from RTP and control injected rats at 1 week and 1 month. In both RTP and control injected retinas GFAP positive cells were predominantly located in the retinal ganglion cell layer (Figure 5(G–J)), suggesting neither control nor RTP injection induced lasting glial activation. There was no appreciable difference in the extent of GFAP immunoreactivity between control and RTP injected eyes.

As thinning of the retina is an indicator of retinal degeneration, the thickness of the ganglion cell layer (GCL), inner plexiform layer (IPL), inner nuclear layer (INL), outer plexiform layer and (OPL) and outer nuclear layer (ONL) was measured and normalized to the total retinal thickness. There were no differences in thickness in any retinal layers (Figure 5(L,M)) at 1 week (*p* = .06–.42, *n* = 5) or 1 month (*p* = .14–.69, *n* = 5) following RTP injection.

The number of apoptotic retinal cells (as measured by TUNEL, Figure 5(K) was also evaluated at 1 week and 1 month after control and RTP injections. TUNEL positive apoptotic cells were rarely seen and there was no difference in the number of these cells between control and RTP treatment at 1 week (*p* = .67, *n* = 5) and 1 month (*p* = .19, *n* = 5). TUNEL positive apoptotic cells were confirmed in positive control retinal sections pretreated with DNase to induce DNA strand break, prior to TUNEL labeling procedures (data not shown).

### RTP injection does not affect retinal function

**Figure 6.**
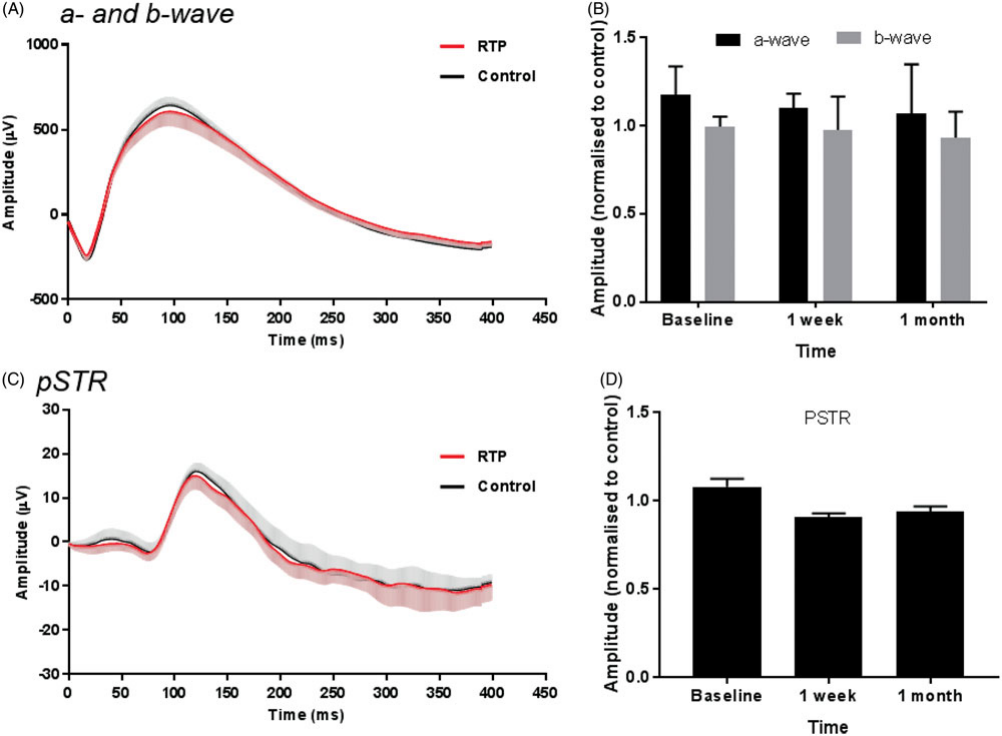
Retinal function of rats receiving intravitreal RTP or control injections. Electroretinography was performed at (*n* = 10) baseline and at 1 week (*n* = 5) and 1 month (*n* = 5) after injection. Mean waveforms (control black line, gray areas shows SD, RTP red line) in (A) photopic (2.2 log(cd.s/m^2^)) and (B) scotopic (−4.94 log(cd.s/m^2^)) conditions. Amplitudes (normalized to their contralateral control amplitudes) of a- and b-wave (C) and pSTR (D). Data shown as mean ± SD.

To investigate the effect of RTP injections on retinal function, electroretinography was performed at baseline and at 1 week and 1 month after injection (Figure 6). Photopic a- and b-wave waveforms were analyzed at 2.2 log(cd.s/m^2^) and pSTR waveforms analyzed at −4.94 log(cd.s/m^2^). Functional measurements in RTP-treated eyes were normalized to their own contralateral control eyes to account for any functional loss resulting from the intravitreal injection procedure itself. Over 1 month, there were no significant differences in photoreceptor (one-way ANOVA F = 0.02, *p* = .98) or bipolar cell (one-way ANOVA, F = 0.07, *p* = .93) function between treated and control eyes. Ganglion cell function (pSTR) was also comparable between control and RTP injected eyes (one-way ANOVA, F = 1.17, *p* = .33). No *post-hoc* differences (Dunnett’s multiple comparison test) were noted between time points in any functional measurement.

## Discussion

The present study demonstrates that a reverse thermoresponsive polymer made with biodegradable materials shows *in vitro* and *in vivo* biocompatibility and preliminary evidence of therapeutically-appropriate release profiles *in vitro* for one of two commonly used anti-VEGF drugs.

The *in-vitro* release kinetics for both aflibercept and bevacizumab were conducted at 30% polymer concentration and 1.8% drug content. The extended release study was carried out over 183 days at physiological temperature (37 °C), and with the aid of a shaker to simulate the constant movements of the eye. At each measurement point, half of the PBS solution was sampled and replaced to simulate the natural removal of the active ingredient from the eye, which also allowed maximum concentration effects to be built into the experimental method. Both bevacizumab and aflibercept were observed to have broadly similar release mechanisms, composed of two phases of release: an initial diffusion-controlled phase where the loosely bound drug diffuses from the polymer gel, followed by a degradation-controlled release phase as the polymer degrades. Bevacizumab was seen to have a significantly higher burst release during the initial 14 day diffusion phase. Approximately 67% of bevacizumab was released during the first 14 days as compared to 10% of aflibercept for the same period. In total approximately 95% of bevacizumab and 25% of aflibercept was released over the 183 day period. Molecular weight, hydrodynamic radius, and hydrogel binding affinity, among other factors, are known to affect release rates of proteins from hydrogels (Vermonden et al., 2012; Osswald and Kang-Mieler, 2016), and the difference in release rates is likely due to differences between aflibercept and bevacizumab in one or more of these properties.

While the high initial burst release of bevacizumab can be beneficial in ocular drug release, allowing for rapid increase of intravitreal drug concentration to a therapeutic level, depletion of the drug in the matrix is a potential concern. Nevertheless, if the rate of release of the drug from the polymer gel during the degradation phase can counteract the natural rate of removal of the drug from the eye, the current profile would, in principle, be well suited for drug delivery *in vivo*. Achieving a higher initial drug loading, together with an increase in the hydrophilicity of the polymer matrix, through changes to the polymer architecture or additives, would be needed to minimize burst release and prolong the window of release *in vivo*.

In contrast, aflibercept release from the current polymer-drug combination seems to be very slow, with only 25% of the loaded drug released over 183 days. In this case, decreasing the hydrophilicity of the medium may allow greater diffusion over time. An increased drug loading may also be favorable to improve the initial build-up of the drug to therapeutic levels within the vitreous body. It is important to note that the *in vitro* drug-release study performed in this work is illustrative only and further studies are needed to further characterize release kinetics *in vitro* and *in vivo*.

Based on more than 50 years of research and currently used medical materials in devices, polyesters of glycolic, lactic and caprolatone co-polymers are known to be completely degradable into nontoxic monomers and safely cleared through normal excretion pathways. Thus, as this RTP differs from known nontoxic biodegradable co-polymers in architecture but not in chain composition, it was assumed to be completely degradable to nontoxic monomers, and as such was predicted to have no harmful effects *in vitro* or *in vivo*. Our assessments of safety and biocompatibility of the polymer bore this out in cell studies *in vitro* and in rat eyes *in vivo*. No adverse effects of the RTP were observed on cell viability *in vitro* using human (SH5Y) and rat (R12) cell lines^Figure 3.^. Furthermore the intravitreal injection of RTP into rat eyes was well tolerated with no evidence of associated inflammation or elevated intraocular pressure.

Electroretinogram measurements were unchanged across the course of the study and comparable for RTP and control injected eyes. The amplitudes of a-wave, b-wave and pSTR (measurements of photoreceptor, bipolar cell and ganglion cell function, respectively) in RTP treated eyes were normalized to contralateral sham eyes in order to account for any loss of function caused by mechanical effects of the injection procedure itself. These functional parameters have been shown to be sensitive to various forms of retinal insult, including inflammation (Tremblay et al., 2013), elevated IOP (Bui et al., 2005) and toxicity (Fielden et al., 2015). At both 1 week and 1 month after injection, we saw no significant changes in any functional parameters, indicating that the presence of the polymer in the vitreous does not have a deleterious effect on retinal function.

Moreover, *in vivo* imaging of the RTP-injected eyes shows no evidence of ocular pathology, with no signs of intraocular hemorrhage, inflammation, cataract or retinal detachment. There is some expected obscuration of retinal detail immediately post-injection (Figure 4(E)) – a result of minor opacification of the ocular media due to the anesthesia and injection procedure – though this is transient and retinal imaging shows no abnormalities at 1 week and 1 month after injection.

Microscopic examination of the retinas showed that RTP injection did not have an adverse effect on the morphology of the retinal layers and did not cause an inflammatory response. Microglia and macrophages are inflammatory cells in the retina and their accumulation in the retina is indicative of inflammation (Bui et al., 2005). RTP-injected retinas showed comparable number of macrophages and microglia to the control treated retinas, and importantly these inflammatory cells show a predominantly inactive, ramified morphology.

Glial activation is an indicator of retinal stress, and during inflammatory events the associated expression of GFAP is demonstrated in all retinal layers including ganglion cell layer and inner and outer retinal layers (Tuccari et al., 1986; Fernandez-Sanchez et al., 2015). GFAP-positive cells were predominantly seen in the retinal ganglion cell layer of RTP and control injected retinas indicating minimal glial activation. Importantly, no GFAP-positive cell processes were seen extending through the retinal layers, indicating that Müller cells were not being activated. Moreover, the number of apoptotic cells and the thickness of the retinal layers were both similar between RTP and control injected eyes. These observations suggest that RTP does not cause inflammation or gliosis in retinal tissues up to one month post-injection, suggesting that it may be compatible for use *in vivo*.

A limitation of this study is that we have not investigated the stability of the released protein over time, nor have we studied drug release from the polymer *in vivo*. We have shown that the reverse thermoresponsive polymer tested here possesses a number of important physical and safety characteristics that make it a candidate for use as an intravitreal slow-release drug delivery vehicle. Further work is needed to ascertain the stability and efficacy of drugs delivered in this way.

## Conclusions

We have shown that the RTP is well-suited for use as sustained drug delivery vehicle in the eyes of adult rats. The drug and vehicle can be mixed at room temperature and injected in liquid form into the eye, using conventional intravitreal injection technique, where they combine to form a gel capable of delayed anti-VEGF drug release. This work serves as the basis for further studies to explore the use of thermoresponsive polymers in the treatment of people with ocular disorders such as AMD and diabetic macular edema who require ongoing anti-VEGF therapy.

## Supplementary Material

UDRD_2018_0441_Supplementary_Materials.docx
