# Supplementary material for: A biocompatible reverse thermoresponsive polymer for ocular drug delivery
Source: Drug Deliv. 2019 Mar 24;26(1):343–53. doi: 10.1080/10717544.2019.1587042 (PMC6442223; doi:10.1080/10717544.2019.1587042)
Supplement: UDRD_2018_0441_Supplementary_Materials.docx [file IDRD_A_1587042_SM8863.docx]

**Supplementary Materials**

| **Table S1 -** aqueous polymer:bevacizumab solution reagents | | |
| --- | --- | --- |
| **Reagent** | **Mass (mg)** | **Percentage (%)** |
| Polymer | 428.57 | 30 |
| Bevacizumab | 25 | 1.8 |
| Bevacizumab solution | 975 | 68.3 |
| PBS | 0 | 0 |
| **Total** | 1428.57 | 100 |

| **Table S2 -** aqueous polymer:aflibercept solution reagents | | |
| --- | --- | --- |
| **Reagent** | **Mass (mg)** | **Percentage (%)** |
| Polymer | 666.67 | 30 |
| Aflibercept | 40 | 1.8 |
| Aflibercept solution | 960 | 43.2 |
| PBS | 556 | 25 |
| **Total** | 2222.67 | 100 |

| **Table S3** – ratio of components in polymer synthesis | | |
| --- | --- | --- |
| **Component** | **MW (g)** | **ratio** |
| PE-LA:CL | 2000 | 1 |
| HDI | 168.2 | 3.364 |
| PEG (350) | 350 | 1.05 |

| **Table S4** - GPC results | |  |  |  |  |  |  |
| --- | --- | --- | --- | --- | --- | --- | --- |
| **Retention time** | **Adjusted RT** | **Mn** | **Mw** | **MP** | **Mz** | **Mz+1** | **Polydispersity** |
| 29.869 | 29.869 | 2766 | 3469 | 3356 | 4266 | 5110 | 1.254034 |


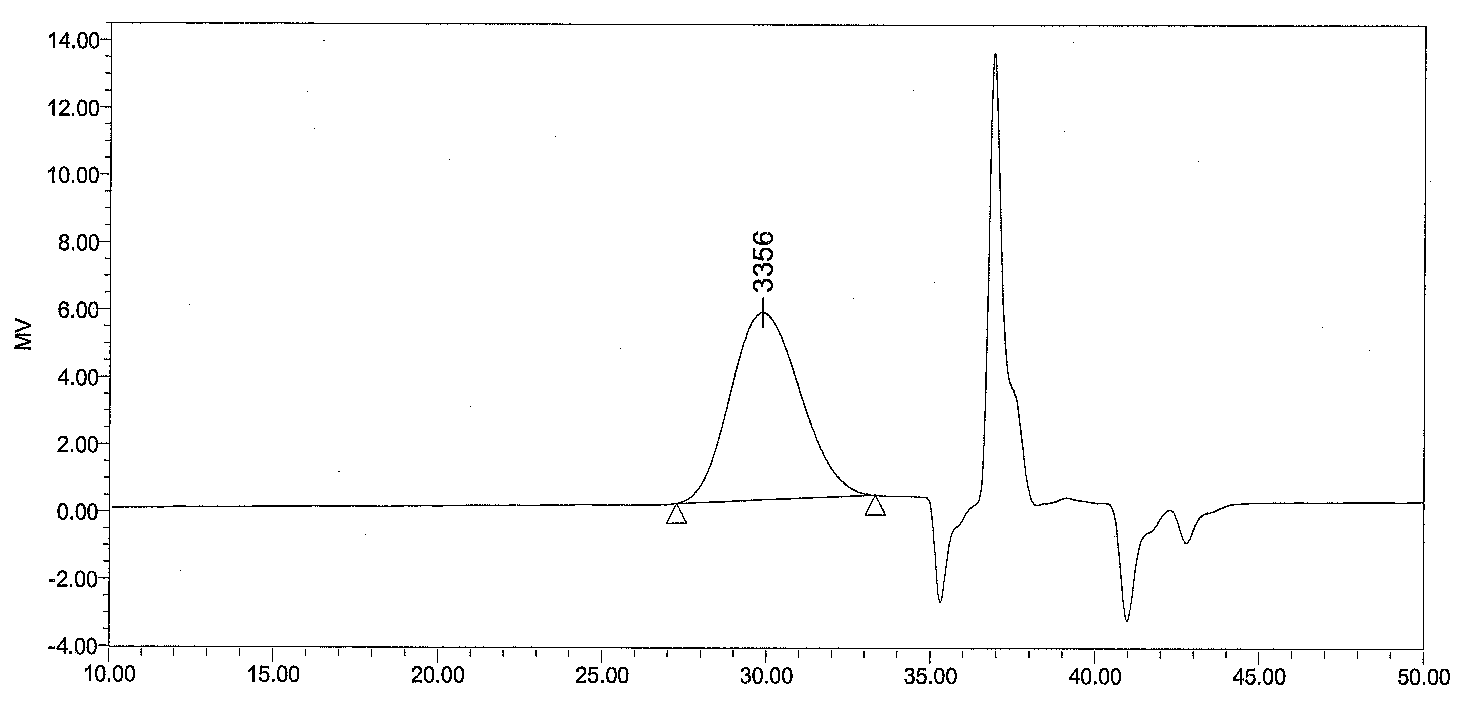


Time (minutes)

MV


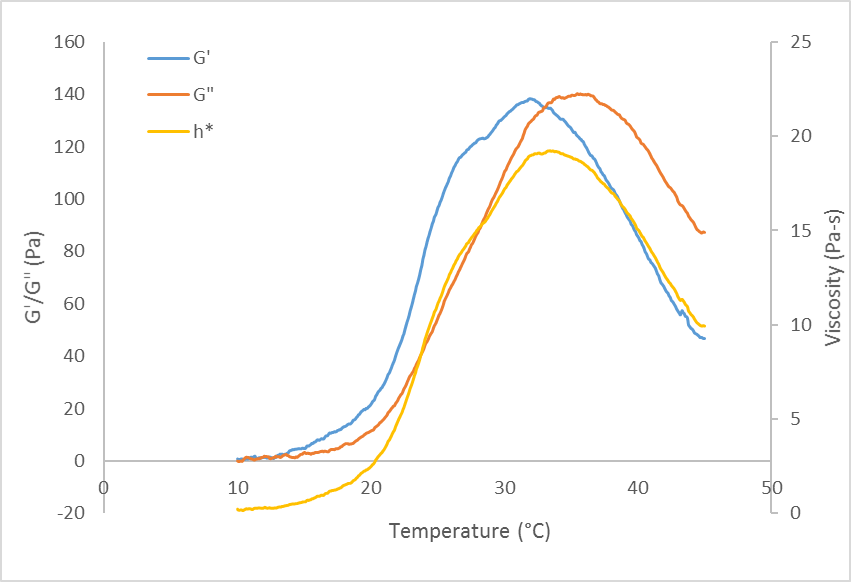


A

B

**Figure S1** Gas phase chromatography and rheology of the reverse thermoresponsive polymer. A shows GPC curve, B shows changes in G’ (blue), G” (orange) and h* (yellow) across a range of temperatures from 10 to 45°C.
